# Galactose Ingested with a High-Fat Beverage Increases Postprandial Lipemia Compared with Glucose but Not Fructose Ingestion in Healthy Men

**DOI:** 10.1093/jn/nxaa105

**Published:** 2020-04-16

**Authors:** Jonathan Watkins, Aaron Simpson, James A Betts, Dylan Thompson, Adrian Holliday, Kevin Deighton, Javier T Gonzalez

**Affiliations:** Department for Health, University of Bath, Bath, United Kingdom; Institute for Sport, Physical Activity & Leisure, Leeds Beckett University, Leeds, United Kingdom; Department for Health, University of Bath, Bath, United Kingdom; Department for Health, University of Bath, Bath, United Kingdom; Institute for Sport, Physical Activity & Leisure, Leeds Beckett University, Leeds, United Kingdom; Institute for Sport, Physical Activity & Leisure, Leeds Beckett University, Leeds, United Kingdom; Department for Health, University of Bath, Bath, United Kingdom

**Keywords:** sugar, metabolism, lipids, fat metabolism, triacylglycerols

## Abstract

**Background:**

Fructose ingestion with a high-fat beverage increases postprandial lipemia when compared with glucose. It is unknown whether other sugars, such as galactose, also increase postprandial lipemia.

**Objectives:**

The objective was to assess whether galactose ingestion within a high-fat beverage increases postprandial lipemia relative to glucose or fructose.

**Methods:**

Two experiments were conducted, which contrasted different test drinks under otherwise standardized conditions. In Experiment 1, 10 nonobese men (age: 22 ± 1 y; BMI, 23.5 ± 2.2 kg/^2^) ingested either galactose or glucose (0.75 g supplemented carbohydrate per⋅kilogram body mass) within a high-fat test drink (0.94 g fat per kilogram body mass). In Experiment 2, a separate group of 9 nonobese men (age: 26 ± 6 y; BMI: 23.5 ± 2.6 kg/m^2^) ingested either galactose or fructose (identical doses as those in Experiment 1) within the same high-fat test drink. Capillary blood was sampled before and at frequent intervals after ingestion of the test drinks for a 300-min period to determine plasma triacylglycerol, glucose, lactate, nonesterified fatty acid, and insulin concentrations. Paired *t* tests and 2-way, repeated-measures ANOVA were used to compare conditions within each experiment.

**Results:**

The incremental AUC for triacylglycerol was greater following galactose ingestion compared with glucose (127 ± 59 compared with 80 ± 48 mmol⋅L^−1^ × 300 min, respectively; *P *= 0.04) but not compared with fructose (136 ± 74 compared with 133 ± 63 mmol⋅L^−1^ ×300 min, respectively; *P *= 0.91). Plasma lactate concentrations also increased to a greater extent with galactose compared with glucose ingestion (time–condition interaction: *P* < 0.001) but not fructose ingestion (time–condition interaction: *P *= 0.17).

**Conclusions:**

Galactose ingestion within a high-fat beverage exacerbates postprandial lipemia and plasma lactate concentrations compared with glucose but not fructose in nonobese men. These data suggest that galactose metabolism may be more similar to fructose than to glucose, providing a rationale to reassess the metabolic fate of galactose ingestion in humans. This trial was registered at clinicaltrials.gov as NCT03439878.

## Introduction

Plasma triacylglycerol concentrations (lipemia) are a primary risk factor for cardiovascular disease (CVD), with Mendelian randomization supporting a causal role of plasma triacylglycerol concentrations in coronary artery disease (1). Postprandial triacylglycerol concentrations are thought to provide a better representation of metabolic health than fasting triacylglycerol concentrations for at least 2 reasons (2): *1*) The fed state captures the total amount of atherogenic triacylglycerol-rich lipoproteins in plasma (from both hepatic and intestinal origin) and *2*) the postprandial state predominates most of the 24-h cycle for the majority of populations in developed countries (3). Postprandial, as opposed to fasting, triacylglycerol concentrations therefore better reflect habitual exposure to atherogenic lipoproteins directly involved in CVD. As such, individuals with nonfasting triacylglycerol concentrations >3.5 mmol⋅L^−1^ (sampled between 1 and 8 h after a meal) have a >3-fold increased CVD mortality compared with those displaying triacylglycerol concentrations <1 mmol⋅L^−1^ (2, 4).

Primary nutritional factors that acutely modulate postprandial lipemia include the type and amount of fat in a meal and the type and amount of carbohydrate that is coingested. Ultimately, plasma triacylglycerol concentrations reflect the rate of triacylglycerol appearance (primarily from hepatically derived VLDL triacylglycerol and intestinally derived chylomicron triacylglycerols) relative to the rate of triacylglycerol disappearance [primarily into adipose tissue and skeletal muscle via lipoprotein lipase (LPL) action]. The type of carbohydrate ingested can further modulate postprandial lipemia via most of these pathways. For example, fructose ingestion stimulates hepatic de novo lipogenesis (potentially via lactate production as a precursor) and glycogen synthesis while suppressing fatty acid oxidation compared with glucose ingestion (5, 6), which would result in greater net hepatic triacylglycerol synthesis and VLDL production (7). In addition, fructose produces a lower insulin response than glucose (5). In healthy individuals, acute increases in insulinemia suppress lipolysis and stimulate LPL activity in adipose tissue (8) while directly suppressing VLDL secretion by the liver (9). Accordingly, the lower insulin response to fructose ingestion is also likely to be a major mechanism explaining the increase in postprandial lipemia via less suppression of hepatic VLDL secretion (9), less adipose clearance of triacylglycerols due to lower LPL activity (10), and higher hepatic nonesterified fatty acid (NEFA) availability [which contributes to VLDL production (9)].

Galactose is the distinctive monosaccharide of the milk sugar, lactose (a glucose–galactose disaccharide). Interestingly, some key metabolic responses to galactose ingestion appear to mirror those of fructose ingestion, which may be important for the regulation of postprandial lipemia. For example, galactose ingestion results in lower plasma glucose and insulin responses than glucose (11), and galactose ingestion potently stimulates hepatic glycogen synthesis (12, 13). This raises the possibility that galactose metabolism is more similar to fructose than to glucose and may therefore exaggerate postprandial lipemia compared with glucose ingestion, via a reduction in insulinemia and/or direct changes in hepatic metabolism. The only randomized, controlled study in humans to measure triacylglycerol responses to galactose supplementation showed that fasting triacylglycerol concentrations were increased by ∼30% with 4 d of galactose compared with glucose supplementation (14). However, to date, no study has assessed the acute postprandial triacylglycerol responses to galactose ingestion compared with glucose or fructose.

To this end, the aim of this study was to assess the acute effect of galactose on postprandial lipemia compared with glucose (Experiment 1) and fructose (Experiment 2). We hypothesized that galactose ingestion would increase plasma triacylglycerol concentrations compared with glucose but not fructose ingestion.

## Methods

This study comprised 2 experiments, each conducted with identical outcome measures and inclusion/exclusion criteria. Experiment 1 compared galactose with glucose, and Experiment 2 compared galactose with fructose.

### Experimental design

Both experiments were conducted as single-blind (participant-blinded), randomized crossover trials, each with 2 arms. Following written, informed consent, participants visited the laboratories on 2 occasions, each with a washout period of 7–28 d. Experiment 1 was conducted at the University of Bath, and Experiment 2 was conducted at Leeds Beckett University. The experiments were conducted with different groups of participants, but the participants were recruited with identical inclusion/exclusion criteria. Once participants were recruited to each separate experiment, they were randomly assigned to a treatment sequence. The study protocols were approved by the University of Bath Research Ethics Approval Committee for Health and the Leeds Beckett Research Ethics Committee for Experiment 1 and Experiment 2, respectively. All procedures were carried out in accordance with the Declaration of Helsinki.

### Participants

For both experiments, the aim was to recruit 10 nonobese [BMI (in kg/m^2^) <30], healthy men from the local area. Inclusion criteria included age 18–35 y, no history of metabolic disease, and free from allergies of milk and cream. For Experiment 2, 1 participant was excluded following completion data analysis after it became clear that his BMI was >30, which was the prespecified inclusion criteria (BMI of this individual was 32). Importantly, the inclusion/exclusion of this participant does not affect any of the statistical interpretations of any of the outcome measures (data not shown). Accordingly, *n* = 10 for Experiment 1 and *n* = 9 for Experiment 2. Participant characteristics are reported in **Table 1**.

**TABLE 1:** Participant characteristics

	Experiment 1 (*n* = 10)	Experiment 2 (*n* = 9)
Age, y	22 ± 1	26 ± 6
Stature, cm	180 ± 6	181 ± 7
Body mass, kg	76 ± 5	78 ± 14
BMI, kg/m^2^	23.5 ± 2.2	23.5 ± 2.6

Data are means ± SDs.

### Trial days

For 48 h prior to each laboratory trial day, participants standardized their diet and physical activity and refrained from alcohol consumption and smoking. Participants also avoided caffeine for 24 h prior to trials and arrived at the laboratory between 08:30 and 08:45 in an overnight-fasted state (12-h fast). Water consumption was permitted ad libitum, recorded on first condition, and replicated on the second condition.

Upon arrival at the laboratory, body mass was determined to the nearest 0.1 kg (424 Sliding Beam Column scale; Weylux) after voiding; participants wore only light clothing. Height was measured to the nearest 0.1 cm using a stadiometer (Seca). A baseline capillary blood sample was then taken within 15 min prior to ingestion of the test drink. Participants consumed the test drink within 5 min. A stop clock was started upon ingestion of the first mouthful, with further capillary blood samples taken at 30, 60, 90, 120, 180, 240, and 300 min to determine plasma metabolite and insulin responses to the test drinks.

### Test drink composition

The test drinks provided 0.94 g fat per kilogram body mass and 0.75 g supplemental carbohydrate per kilogram body mass. They were composed of cream and whole-fat milk, supplemented with galactose (Galaxtra; Solace Nutrition), dextrose (Myprotein), or fructose (Holland & Barrett), all flavored with 0.5 mL of chocolate/vanilla flavor droplets (Myprotein Flavdrops). The composition of test meals is given in **Table 2**. The rationale for the dose of fat was to produce a robust triacylglyceridemic response (15), and that for the dose of carbohydrate was to broadly replicate prior work on fructose ingestion (5). We normalized the doses to body mass in an attempt to standardize the relative nutrient exposure across participants.

**TABLE 2:** Test drink composition

	Experiment 1 (*n* = 10)		Experiment 2 (*n* = 9)	
	Glucose	Galactose	Fructose	Galactose
Energy, kcal·kg^−1^	13	13	13	13
Fat, g·kg^−1^	0.94	0.94	0.94	0.94
Lactose, g·kg^−1^	0.17	0.17	0.17	0.17
Glucose, g·kg^−1^	0.75	0	0	0
Galactose, g·kg^−1^	0	0.75	0	0.75
Fructose, g·kg^−1^	0	0	0.75	0
Protein, g·kg^−1^	0.12	0.12	0.12	0.12

### Blood sampling and analyses

To stimulate peripheral blood flow, participants placed their hand in a bowl of warm (∼40°C) water 5 min prior to each capillary blood sample. Blood samples were collected via finger prick (Lancets Accu-Check Safe T Pro Plus; Roche Diabetes Care) in a 300-μL microvette capillary tube (Microvette CB 300 K2E; Sarstedt). Capillary sampling is preferable to venous sampling for postprandial concentrations of glucose and insulin because peripheral tissues extract glucose and lactate to a variable extent in the postprandial state. This leads to lower concentrations in venous blood compared with arterial/arterialized blood; due to the variable nature of this reduction, simple correction factors are not possible (16, 17). Capillary blood has been shown to be representative of arterialized blood under a variety of metabolic conditions (16) and therefore is preferable to venous blood sampling for determining the peripheral exposure to metabolites and hormones.

The collected capillary samples were centrifuged (Hareus Biofuge Pico; DJB Labcare) at 9503 × *g* for 10 min at room temperature. The extracted plasma samples were aliquoted into 0.5-mL tubes (model 5810; Eppendorf) and stored in a freezer at −80°C before later analysis. Plasma triacylglycerol and NEFA concentrations were measured in duplicate and were assayed using enzymatic colorimetric techniques (Wako Pure Chemical Industries). Interplate CVs were <8.7% and <5.9% for triacylglycerol and NEFA, respectively. Plasma insulin concentrations were analyzed in singular using a solid-phase 2-site enzyme immunoassay (Mercodia Insulin ELISA; Mercodia). Plasma glucose and l-lactate concentrations were analyzed in singular using an automated analyzer (YSI 2300 Stat Plus; YSI). Due to a lack of sufficient sample for 1 participant, plasma lactate concentrations are *n* = 9 for both experiments.

### Calculations and statistical analyses

The primary outcome for the current study was the incremental AUC (iAUC) for plasma triacylglycerol concentrations. Plasma glucose, insulin, NEFA, and lactate concentrations were secondary outcome measures. The sample size was based on prior work comparing fructose with starch (as a glucose-based carbohydrate) ingestion within a high-fat beverage on postprandial triacylglycerol concentrations (18). The time-averaged iAUC for triacylglycerol concentrations following fructose ingestion was 609 ± 203 μmol·L^−1^ compared with 379 ± 191 μmol·L^−1^ following starch ingestion. Based on this effect size (*d* = 1.17), 10 participants should provide >90% power to detect an effect of a similar magnitude with an α level of 0.05 when using a 2-tailed, paired *t* test. Data are presented as means ± SDs, unless otherwise stated. The variance bars on figures are presented as means with 95% CIs. Incremental AUC was calculated for triacylglycerol, glucose, insulin, and lactate concentrations using the trapezoid method, ignoring values less than the baseline (19). The total AUC was calculated for NEFA concentrations because these were suppressed below baseline following ingestion of the test drink. Paired differences were checked for normal distribution by the Shapiro–Wilk normality test prior to analysis (with *P* ≤ 0.05 being accepted as significant). Based on these analyses, all data were deemed not to deviate significantly from a normal distribution. Paired *t* tests were employed to assess differences between non-time-dependent variables, whereas differences between conditions in time-dependent variables (plasma triacylglycerol, insulin, NEFA, glucose, and lactate concentrations) were analyzed using a 2-way repeated-measures ANOVA (condition–time). A 2-tailed *P* value of ≤0.05 was considered statistically significant. The magnitude of difference between conditions was also expressed via Cohen's *d* effect size and is presented where small (*d* = 0.2–0.5), moderate (*d* = 0.5–0.8), or large (*d* > 0.8) effects were observed. Out of a possible total of 3040 data points, 18 data points (0.59%) were missing due to a lack of sufficient plasma sample for analysis. When this was the case, the means of samples on each side of this time point were taken (e.g., for a missing 30-min sample, the means of 15-min and 45-min samples were used). Results were analyzed using Microsoft Excel version 15.26 and GraphPad Prism version 8.0.2.

## Results

### Plasma triacylglycerol concentrations

**FIGURE 1:**
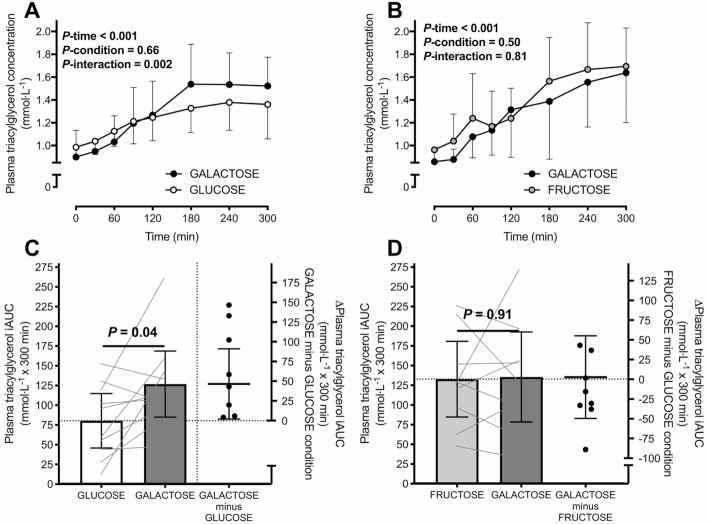
Plasma triacylglycerol concentrations (A, B) and iAUCs (C, D) in healthy, nonobese men following ingestion of a high-fat beverage containing galactose compared with glucose (A, C) or fructose (B, D) in 2 separate experiments. Right-hand *y* axes represent the difference between conditions (within each experiment) for plasma triacylglycerol iAUC. Data are means ± 95% CIs; *n* = 10 (A, C) and *n* = 9 (B, D) nonobese men. Assessed by 2-way, repeated-measures ANOVA. Paired 2-tailed *t* tests were used to compare the iAUC between conditions. iAUC, incremental area under the curve.

In Experiment 1, no differences were detected in fasting plasma triacylglycerol concentrations between conditions (**Table 3**). Following ingestion of the test drinks, plasma triacylglycerol concentrations increased during the postprandial period in both conditions (time effect, *P* < 0.001). The increase in plasma triacylglycerol concentrations was greater following galactose compared with glucose ingestion (*P*-interaction = 0.002; **Figure 1**A). The plasma triacylglycerol iAUC was ∼60% greater with galactose compared with glucose ingestion with a moderate to large effect size (*P *= 0.04; Figure 1C; *d* = 0.75).

**TABLE 3:** Fasting plasma triacylglycerol, glucose, insulin, NEFA, and lactate concentrations in healthy, nonobese men prior to ingestion of a high-fat beverage containing glucose, galactose, or fructose in 2 separate experiments

	Experiment 1 (*n* = 10)			Experiment 2 (*n* = 9)		
	Glucose	Galactose	*P*	Fructose	Galactose	*P*
Plasma triacylglycerol concentration, mmol·L^−1^	0.99 ± 0.14	0.90 ± 0.33	0.270	0.96 ± 0.29	0.86 ± 0.26	0.408
Plasma glucose concentration, mmol·L^−1^	5.06 ± 0.25	5.06 ± 0.38	0.984	5.25 ± 0.22	5.34 ± 0.30	0.402
Plasma insulin concentration, mmol·L^−1^	32 ± 13	31 ± 12	0.749	38 ± 18	38 ± 12	0.904
Plasma NEFA concentration, mmol·L^−1^	0.40 ± 0.14	0.38 ± 0.11	0.618	0.29 ± 0.07	0.35 ± 0.10	0.209
Plasma lactate concentration, mmol·L^−1^	0.92 ± 0.17	0.89 ± 0.21	0.713	1.35 ± 0.49	1.41 ± 0.46	0.646

Data are means ± SDs. *P* values represent 2-tailed, paired *t* tests. NEFA, nonesterified fatty acid.

In Experiment 2, no differences were detected in fasting plasma triacylglycerol concentrations between conditions (Table 3). Following ingestion of the test drinks, plasma triacylglycerol concentrations increased during the postprandial period in both conditions (time effect, *P* < 0.001) but did not differ between galactose and fructose ingestion (*P*-interaction = 0.81; Figure 1B). There was no difference in the plasma triacylglycerol iAUC between galactose and fructose ingestion, and there was a negligible effect size (*P *= 0.91; Figure 1D; *d* = 0.04).

### Plasma glucose and insulin concentrations

**FIGURE 2:**
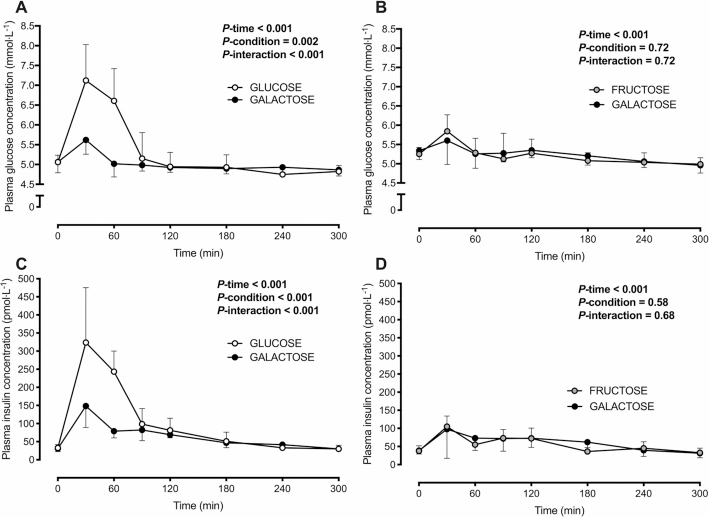
Plasma glucose (A, B) and insulin (C, D) concentrations in healthy, nonobese men following ingestion of a high-fat beverage containing galactose compared with glucose (A, C) or fructose (B, D) in 2 separate experiments. Data are means ± 95% CIs; *n* = 10 (A, C) and *n* = 9 (B, D) nonobese men. Assessed by 2-way, repeated-measures ANOVA.

In Experiment 1, no differences were detected in fasting plasma glucose or insulin concentrations between conditions (Table 3). Following ingestion of the test drinks, plasma glucose concentrations initially increased (time effect, *P* < 0.001) to a peak at 45 ± 29 and 39 ± 14 min following glucose and galactose ingestion, respectively (*P *= 0.62). The increase in plasma glucose concentrations was smaller with galactose than with glucose ingestion (*P*-interaction < 0.001; **Figure 2**A, **Table 4**). Similarly, plasma insulin concentrations initially increased (time effect, *P* < 0.001) to a peak at 45 ± 16 and 51 ± 32 min following glucose and galactose ingestion, respectively (*P *= 0.51). The increase in plasma insulin concentrations was smaller with galactose than with glucose ingestion (*P*-interaction < 0.001; Figure 2C).

**TABLE 4:** Postprandial iAUC of glucose, insulin, NEFA, and lactate concentrations in healthy, nonobese men following ingestion of a high-fat beverage containing glucose, galactose, or fructose in 2 separate experiments

	Experiment 1 (*n* = 10)			Experiment 2 (*n* = 9)		
	Glucose	Galactose	*P*	Fructose	Galactose	*P*
Glucose iAUC, mmol·L^−1^ × 300 min	123 ± 51	46 ± 47	0.004	44 ± 64	21 ± 17	0.31
Insulin iAUC, mmol·L^−1^ × 300 min	21 ± 9	10 ± 4	0.001	6 ± 4	8 ± 6	0.53
NEFA AUC, mmol·L^−1^ × 300 min	100 ± 21	95 ± 14	0.35	88 ± 23	97 ± 20	0.29
Lactate iAUC, mmol·L^−1^ × 300 min	51 ± 30	208 ± 35	<0.001	212 ± 93	222 ± 133	0.87

Data are means ± SDs. *P* values represent 2-tailed, paired *t* tests. iAUC, incremental AUC; NEFA, nonesterified fatty acid.

In Experiment 2, no differences were detected in fasting plasma glucose or insulin concentrations between conditions (Table 3). Following ingestion of the test drinks, plasma glucose initially increased (time effect, *P* < 0.001) to a peak at 37 ± 20 and 57 ± 51 min following fructose and galactose ingestion, respectively (*P *= 0.30). Plasma glucose concentrations did not differ following galactose compared with fructose ingestion (*P*-interaction = 0.72; Figure 2B, Table 4). Similarly, plasma insulin concentrations initially increased (time effect, *P* < 0.001) to a peak at 50 ± 34 and 130 ± 89 min following fructose and galactose ingestion, respectively (*P *= 0.032), and plasma insulin concentrations did not differ following galactose compared with fructose ingestion (*P*-interaction = 0.68; Figure 2D).

### Plasma NEFA and lactate concentrations

**FIGURE 3:**
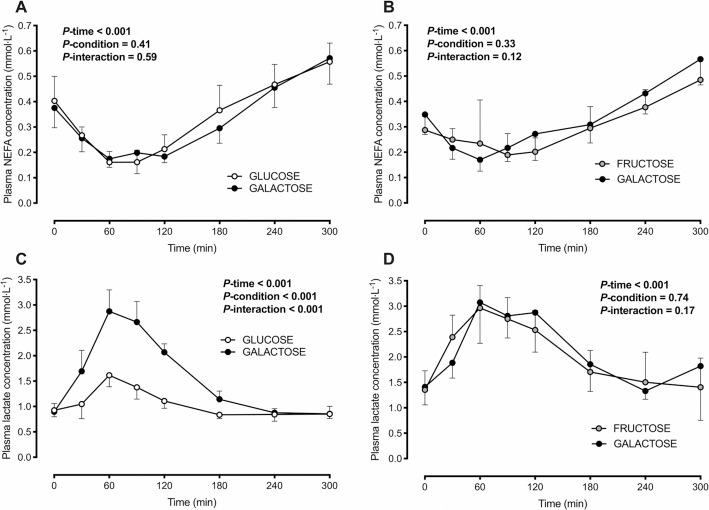
Plasma NEFA (A, B) and lactate (C, D) concentrations in healthy, nonobese men following ingestion of a high-fat beverage containing galactose compared with glucose (A, C) or fructose (B, D) in 2 separate experiments. Data are means ± 95% CIs; *n* = 10 (A) and *n* = 9 (B–D) nonobese men. Assessed by 2-way, repeated-measures ANOVA. NEFA, nonesterified fatty acid.

In Experiment 1, no differences were detected in fasting plasma NEFA or lactate concentrations between conditions (Table 3). Following ingestion of the test drinks, plasma NEFA concentrations declined during the initial postprandial period in both conditions (time effect, *P* < 0.001). The decline in plasma NEFA concentrations did not differ between galactose and glucose (*P*-interaction = 0.59; **Figure 3**A, Table 4). Plasma lactate concentrations initially increased in both conditions (time effect, *P* < 0.001). The increase in plasma lactate concentrations was ∼4-fold greater with galactose than with glucose ingestion (*P*-interaction < 0.001; Figure 3C, Table 4).

In Experiment 2, no differences were detected in fasting plasma NEFA or lactate concentrations between conditions (Table 3). Following ingestion of the test drinks, plasma NEFA concentrations declined during the initial postprandial period in both conditions (time effect, *P* < 0.001). The decline in plasma NEFA concentrations did not differ between galactose and fructose ingestion (*P*-interaction = 0.12; Figure 3B, Table 4). Plasma lactate concentrations initially increased in both conditions (time effect, *P* < 0.001) and did not differ following galactose or fructose ingestion (*P*-interaction = 0.17; Figure 3D, Table 4).

## Discussion

This study is the first to demonstrate that acute ingestion of a large (∼58 g) dose of galactose within a high-fat beverage modestly increases postprandial triacylglycerol concentrations compared with glucose but not fructose. Furthermore, galactose ingestion resulted in higher plasma lactate but lower glucose and insulin concentrations compared with glucose but not fructose ingestion. Collectively, these data suggest that galactose produces a metabolic milieu that is more similar to fructose than to glucose ingestion, providing a rationale to reassess the metabolic consequences of milk sugars in humans.

The modest increase in plasma triacylglycerol concentrations seen with ∼58 g of galactose compared with glucose ingestion could be due to increased triacylglycerol appearance rates and/or decreased plasma triacylglycerol clearance. Insulin is a key hormone regulating triacylglycerol metabolism in the postprandial state. In the current study, insulinemia and glycemia during the first 90 min of the postprandial state following galactose ingestion was less than half that seen following glucose ingestion. On the other hand, both insulinemia and glycemia displayed comparable responses following galactose *versus* fructose ingestion. These data are therefore consistent with the idea that the lower plasma glucose concentrations following galactose compared with glucose ingestion stimulated a lower insulinemic response, and that in turn could partly explain the elevated triacylglycerol concentrations.

Lower plasma insulin concentrations could lead to higher triacylglycerol concentrations via a number of mechanisms, including lesser suppression of adipose tissue lipolysis and thus greater hepatic NEFA availability (used for VLDL triacylglycerol production), less direct suppression of VLDL triacylglycerol secretion, and less triacylglycerol clearance by lower activation of LPL in adipose tissue. The first of these potential mechanisms is unlikely to explain the higher triacylglycerol concentrations observed with galactose compared with glucose ingestion in the current study. In healthy individuals, insulin concentrations of ∼34 pmol⋅L^−1^ are sufficient to almost completely inhibit whole-body lipolysis (8). Because plasma insulinemia peaked at >100 pmol·L^−1^ following ingestion of glucose, galactose, or fructose, the suppression of adipose tissue lipolysis is likely to have been maximal under all conditions in the current study, and thus hepatic NEFA availability for VLDL production would not have been altered by galactose compared with glucose ingestion (9). Moreover, the higher lactate concentrations with galactose compared with glucose ingestion could also suppress lipolysis and thereby offset any reduction in insulinemia on NEFA appearance (20), although the effects of lactate on adipose lipolysis in humans are still unclear (21, 22). Nevertheless, plasma NEFA concentrations in the current study did not differ following galactose compared with glucose ingestion, suggesting lipolysis was similarly suppressed in both conditions.

The higher triacylglycerol concentrations with galactose compared with glucose ingestion could therefore be due to less direct suppression of VLDL secretion as a result of lower insulinemia (9). However, there is also evidence that the difference in insulinemia observed in the current study may be insufficient to influence postprandial plasma triacylglycerol concentrations, at least during the initial 180 min of the postprandial period. For example, isomaltulose is composed of identical monomers as sucrose. Due to a slow rate of hydrolysis, isomaltulose ingestion produces lower glycemic and insulinemic responses than sucrose, likely due to slower substrate fluxes (23, 24). The difference in insulinemia with isomaltulose compared with sucrose is comparable to the insulin responses observed in the current study (23), yet no difference in triacylglycerol concentrations was reported with isomaltulose compared with sucrose ingestion with a mixed meal (23). This suggests that the exaggerated triacylglycerol response to galactose ingestion may not solely be due to differences in insulinemia and could be explained by other contributory mechanisms.

Further regulators of triacylglycerol metabolism include hepatic de novo lipogenesis (DNL) and intestinal lipoprotein production, which each could contribute to higher postprandial triacylglycerol concentrations. Fructose ingestion is well known to stimulate hepatic DNL acutely and chronically (7), and it can also stimulate intestinal production of triacylglycerol-rich lipoproteins from enterocytes (25). Although the direct conversion of fructose into fatty acids is not likely to be quantitatively important for postprandial lipemia (5), stimulation of hepatic DNL could be quantitatively important for plasma triacylglycerol concentrations if there is a greater conversion of all DNL precursors (e.g., glucose, fructose, and lactate) into DNL products (fatty acids and glycerol) (7). The acute increase in DNL with fructose ingestion is thought to be due to the rapid and relatively unregulated flux of fructose metabolism in the liver. This leads to an accumulation of triose phosphate, which can be converted into triacylglycerols, lactate, and/or glucose and glycogen (7). Therefore, the increase in lactate concentrations following ingestion of carbohydrates may be reflective of accumulation of triose phosphate and could occur in parallel with DNL. Furthermore, the potent stimulation of glycogen synthesis could also tip the balance of hepatic fatty acid metabolism toward net lipid synthesis (7). The classical view of human galactose metabolism is that the Leloir pathway is the dominant pathway, whereby galactose is ultimately converted into UDP-glucose, and subsequent steps then follow similar pathways to glucose metabolism (26). In the current study, we observed greater increases in plasma lactate concentrations following galactose compared with glucose ingestion and comparable lactate concentrations following galactose compared with fructose ingestion. Because the increase in lactate concentrations with fructose ingestion is thought to be due to the lack of feedback and regulation of hepatic fructose metabolism compared with glucose metabolism (7, 27), the current data suggest that galactose metabolism may be more similar to fructose than to glucose metabolism. When interpreted alongside the evidence that fructose and galactose ingestion both potently stimulate hepatic glycogen synthesis compared with glucose ingestion (12), this raises the possibility that glycogen fluxes could be involved in the postprandial triacylglycerol responses observed in the current study (7) and questions the accuracy of the classical view of galactose metabolism in humans. These data therefore provide a basis for future work to establish the mechanistic effects of galactose on hepatic lipid metabolism in humans.

The current study was conducted in nonobese men; there is a need to understand if galactose ingestion alters postprandial triacylglycerol metabolism in other populations. Given that sex and obesity can each alter the metabolic responses to fructose ingestion (18, 28), future work should establish whether there are sex differences in response to galactose ingestion and whether obesity status alters this response. Due to the measurement of triacylglycerol concentrations alone, it is not possible to establish whether the higher triacylglycerol concentrations were due to an increase in triacylglycerol-rich lipoprotein size or particle number. In addition, the potential mechanisms that may explain the acute increase in postprandial lipemia require elucidation. A further limitation with the current study is that galactose and fructose were provided without adding glucose to the test drinks. This may have implications for translation because galactose and fructose are almost always ingested along with glucose under free-living conditions, which can alter the metabolism of these sugars (29). The dose of galactose ingested in the current study was relatively large (equivalent in sugar content to ∼1.4 L of milk); galactose is unlikely to be commonly consumed in this dose under free-living conditions. Nevertheless, if galactose and fructose exaggerate postprandial lipemia via shared mechanisms, an additive or synergistic effect of galactose and fructose ingestion is possible. This would have consequences for food products containing galactose and fructose in combination, such as sugar-sweetened milkshakes, yoghurts, and ice cream. Finally, note that the current study design only permits comparisons within each experiment and therefore comparisons between experiments are indirect. This limits the ability to directly assess how galactose and fructose differ in regard to glucose ingestion with a high-fat beverage.

In summary, acute ingestion of ∼58 g of galactose with a high-fat beverage increases postprandial lipemia and plasma lactate concentrations compared with glucose but not fructose. These data suggest that galactose metabolism may be more similar to fructose than to glucose metabolism and thereby provide a rationale to reassess the metabolic consequences of galactose ingestion in humans. Furthermore, the similar metabolic response to galactose and fructose ingestion suggests that ingestion of these sugars may produce combined effects on triacylglycerol metabolism, with potential implications for the ingestion of sugar-sweetened dairy products. However, the large doses used in the current study are not reflective of typical dietary intakes; therefore, further studies are needed using more representative amounts of these sugars to determine their effects on postprandial responses.

## Notes

This work was funded, in part, by a Nutrition Society (UK) Summer Studentship.

Author disclosures: JTG has received funding from Arla Foods Ingredients, Kenniscentrum Suiker & Voeding, and Lucozade Ribena Suntory. JAB has received funding from the BBSRC, GlaxoSmithKline, Lucozade Ribena Suntory, Kellogg's, Nestlé, and PepsiCo. All other authors report no conflicts of interest.

Abbreviations used: CVD, cardiovascular disease; DNL, de novo lipogenesis; iAUC, incremental AUC; LPL, lipoprotein lipase; NEFA, nonesterified fatty acid.
